# Anti-Müllerian hormone as a biomarker of ovarian function and spontaneous puberty in Turner syndrome: a systematic review

**DOI:** 10.3389/fendo.2025.1640583

**Published:** 2025-09-15

**Authors:** Bassam Bin-Abbas, Mosleh Jabari

**Affiliations:** ^1^ Department of Pediatrics, King Faisal Specialist Hospital and Research Center, Riyadh, Saudi Arabia; ^2^ Department of Pediatrics, College of Medicine, Imam Mohammad Ibn Saud Islamic University (IMSIU), Riyadh, Saudi Arabia

**Keywords:** Turner syndrome, anti-Müllerian hormone, ovarian function, fertility preservation, spontaneous puberty, premature ovarian insufficiency, ovarian reserve, systematic review

## Abstract

**Background:**

Turner syndrome (TS), caused by complete or partial X chromosome monosomy, often leads to primary ovarian insufficiency (POI) and pubertal delay. Anti-Müllerian hormone (AMH) is a key biomarker of ovarian reserve, but its predictive role in spontaneous puberty and ovarian function in TS remains unclear.

**Methods:**

This systematic review followed PRISMA guidelines and included studies from PubMed, Embase, and Cochrane (2000–2025). Nine studies (865 TS patients, 976 controls) were analyzed. Outcomes included AMH levels in TS versus controls, association with spontaneous puberty, and predictive value for fertility preservation. Risk of bias was assessed using the Newcastle-Ottawa Scale.

**Results:**

TS patients had significantly lower AMH levels than controls (weighted mean differences (WMD): -3.04 ng/mL, 95% CI: -3.26 to -2.83, p < 0.001). Detectable AMH correlated with spontaneous puberty (OR=5.12, 95% CI: 2.87–9.12), particularly in mosaic karyotypes. Subgroup analyses revealed assay variability, with ELISA-based methods detecting low but clinically relevant AMH levels. Sensitivity analyses confirmed robustness, and publication bias was minimal (Egger’s p = 0.283).

**Conclusion:**

AMH is a reliable biomarker for ovarian reserve and spontaneous puberty prediction in TS. Its integration into clinical practice may improve fertility counseling and hormone therapy timing. However, standardized assays and prospective studies are needed to optimize its diagnostic accuracy.

**Systematic review registration:**

https://www.crd.york.ac.uk/prospero/ PROSPERO, identifier CRD420251051633.

## 1 Introduction

Turner Syndrome (TS), resulting from the complete or partial absence of one X chromosome, is the most common sex chromosome abnormality in females, with a prevalence of approximately 1 in 2,000 live births (1). A defining feature of TS is the accelerated depletion of ovarian follicles, beginning as early as 18 weeks of gestation, which frequently results in primary ovarian insufficiency (POI) (2). Although clinical manifestations vary, gonadal dysgenesis, short stature, and pubertal delay or absence due to ovarian failure are among the most commonly observed features (3). Despite early follicular depletion, approximately 30% of girls with TS undergo spontaneous pubertal development, and 2–3% achieve regular menstrual cycles before premature menopause (4, 5). Additionally, spontaneous pregnancies, though rare, have been reported in approximately 2% of women with TS, typically occurring in their early twenties (5, 6). Notably, recent research has identified viable follicles in some TS patients aged 12–13 years who do not yet show signs of puberty, suggesting a disconnect between morphological ovarian findings and clinical presentation (7).

Anti-Müllerian hormone (AMH), secreted by granulosa cells of preantral and small antral follicles, has emerged as a sensitive and non-invasive biomarker of ovarian reserve (8). In healthy females, AMH is low at birth, rises during the first months of life (reflecting ‘mini-puberty’), peaks in late adolescence and young adulthood, and subsequently declines with age until becoming undetectable after menopause. It has been widely used in the evaluation of reproductive function, including in conditions such as polycystic ovary syndrome (PCOS), early ovarian failure, and for predicting ovarian responsiveness in assisted reproductive technologies (9, 10). Low AMH levels are typically associated with diminished ovarian reserve and ovulatory dysfunction (11).

In the context of TS, spontaneous puberty is generally defined by the onset of secondary sexual characteristics—particularly breast development reaching at least Tanner stage B2—occurring without the administration of exogenous estrogen therapy (12). Some studies further consider spontaneous menarche and elevated estradiol levels as indicators of pubertal development (13). According to established pediatric endocrinology guidelines, pubertal onset is expected around the ages of 13 to 14 years (14). Several studies have investigated the potential relationship between serum AMH levels and spontaneous pubertal development in TS patients. However, these findings remain inconsistent due to variability in study designs, small and heterogeneous sample populations, differing criteria for defining spontaneous puberty, and a lack of standardized AMH thresholds specific to TS (15–17). This gap in the literature underscores the need for a systematic review to consolidate and critically evaluate existing evidence on AMH as a biomarker of ovarian function and spontaneous puberty in individuals with TS. By synthesizing the available data, this review aims to clarify AMH’s clinical utility, assess its predictive accuracy, and provide evidence-based recommendations to support decision-making in the management of TS.

This systematic review was guided by the PICO components: The individuals with Turner syndrome were the population (P) of interest. The studied intervention/exposure (I) was the level of AMH in the serum. In the comparison (C), patients with spontaneous puberty and normal ovarian function were contrasted with those who had delayed or missing puberty and reduced ovarian function. Serum AMH levels and the association between ovarian function and spontaneous puberty were the main outcomes (O). According to these PICO elements, the study’s leading review question was: In Turner syndrome patients, how does serum AMH level compare between those with normal ovarian function and spontaneous puberty versus those with impaired ovarian function or delayed or absent puberty?

The aim of this systematic review is to synthesize existing evidence on the relationship between anti-Müllerian hormone (AMH) levels and ovarian function in Turner syndrome (TS), with three key objectives: (1) to determine the correlation between AMH levels and ovarian reserve, (2) to evaluate AMH’s predictive value for spontaneous puberty onset, and (3) to assess how prognostic factors like age, karyotype, and hormonal status influence AMH’s clinical utility. By establishing AMH as a reliable biomarker, this review seeks to provide evidence-based guidance for clinical decision-making regarding pubertal progression and fertility preservation in TS patients.

## 2 Materials and methods

### 2.1 Protocol and registration

This systematic review was conducted in accordance with the rigorous methodological standards outlined in the Preferred Reporting Items for Systematic Reviews and Meta-Analyses (PRISMA) statement (18). The protocol was prospectively registered in the International Prospective Register of Systematic Reviews (PROSPERO; registration number CRD420251051633), where detailed information on the study objectives, eligibility criteria, data extraction methods, and planned analyses is publicly accessible. No deviations from the registered protocol were made during the course of the review. Given that this review exclusively utilized published data and did not involve primary data collection or direct patient contact, ethical approval was waived by the Institutional Review Board of Imam Mohammad Ibn Saud Islamic University (IMSU), Saudi Arabia.

### 2.2 Data sources and search strategy

The search strategy was tailored to each database and was based on the review questions for this systematic review study. A comprehensive literature search was conducted to identify all relevant studies published between January 2000 and April 2025, ensuring the inclusion of the most recent research. The search encompassed Medline (via PubMed), Embase, the Cochrane Library, and primary trial registries (International Clinical Trials Registry Platform, ClinicalTrials.gov, and the EU Clinical Trials Register). In addition, grey literature sources, including conference abstracts and dissertations, were searched to minimize publication bias. No language restrictions were applied; translations were used when necessary.

The search strategy was customized for each database, using Medical Subject Headings (MeSH) for Medline and EMBASE subject headings for other databases. The PICO-formatted search terms focused the PICO components.

The review study employed specific MeSH and Embase terms such as “Turners syndrome” or “primary ovarian insufficiency,” “ovarian function,” “anti-Müllerian hormone,” “clinical trials,” and “serum AMH level.” Boolean operators like “AND” and “OR” were used with the keywords to retrieve relevant and precise contents. To broaden the scope of the screening process for suitable papers, the relevant studies’ bibliographic lists were also examined.

### 2.3 Inclusion and exclusion criteria

The following criteria were used for the eligibility of studies: original studies like cohort studies, randomized controlled trials, and case-controlled studies evaluating the levels of serum anti-Müllerian hormone (AMH) in the prediction of spontaneous puberty and premature ovarian insufficiency (POI) in TS women most commonly as amenorrhea before age 40 years with elevated FSH (>40 IU/L) and low estradiol. The studies that reported (a) reported serum AMH levels in relation to spontaneous puberty or ovarian function; (b) compared serum AMH levels between TS patients and healthy controls; (c) provided usable AMH data, including mean and standard deviation, median and range, or median and interquartile interval. To capture a broad evidence base for this rare condition, case reports, case series, and conference abstracts were considered for narrative synthesis if they contained relevant clinical observations, though priority was given to studies with robust data.

Studies were excluded if they lacked sufficient outcome data for meta-analysis or meaningful interpretation, focused on conditions other than TS, or did not include an appropriate control or comparison group. Studies involving men, transgender individuals, or animal models were also excluded given the genetic and clinical specificity of TS. While lower-evidence study types were generally excluded from quantitative synthesis, their potential insights were acknowledged where applicable.

### 2.4 Data extraction

Study selection was conducted through a two-stage process, beginning with an initial screening of titles and abstracts, followed by a comprehensive full-text review of articles meeting the inclusion criteria. Two independent reviewers (B.B. and M.J.) performed study identification and data extraction, with any discrepancies resolved through discussion or consultation with a third expert in the field. Duplicate records were carefully excluded, retaining only the most recent and complete versions of studies.

Data extraction was carried out using a standardized spreadsheet designed to capture key variables systematically. These included: (a) author and year of publication; (b) study design and methodology; (c) participant demographics such as mean or median age, age range, Turner syndrome phenotype, and karyotype distribution; (d) outcomes including serum AMH levels (mean or median with standard deviation or interquartile range) in relation to age, karyotype, pubertal development status (e.g., Tanner stage, spontaneous versus induced puberty, spontaneous menarche), and ovarian status indicators (e.g., FSH, estradiol, ovarian volume, antral follicle count); (e) details on the AMH measurement method, including assay type, manufacturer, and assay sensitivity to account for inter-study variability; (f) diagnostic criteria used for Turner Syndrome; and (g) level of evidence based on study design and quality assessment tools applied.

Pubertal status definitions varied across studies; where possible, criteria were harmonized using standardized thresholds such as Tanner breast stage ≥ B2 combined with estradiol levels >20 pg/mL to ensure comparability of “spontaneous puberty” classifications (19). AMH values reported in different units were converted to pmol/L using the formula *1 ng/mL = 7.14 pmol/L* to maintain consistency (20). Missing or incomplete outcome data were addressed by contacting study authors when feasible; otherwise, studies with insufficient data were excluded from quantitative synthesis, or imputation methods were applied when appropriate. Patient karyotypes were categorized following the American College of Medical Genetics and Genomics (ACMG) guidelines (21), and established statistical methods were employed to handle missing or inconsistent data, preserving the integrity of the meta-analytic process.

### 2.5 Outcomes

The primary outcomes of this review included the comparison of serum Anti-Müllerian Hormone (AMH) levels between Turner Syndrome (TS) patients and healthy controls, as well as the association between detectable serum AMH levels and spontaneous pubertal development in individuals with TS. Secondary outcomes focused on the predictive value of serum AMH for fertility potential and eligibility for fertility preservation procedures, including oocyte retrieval and ovarian tissue cryopreservation, particularly in mosaic TS patients. Additionally, subgroup analyses were performed to explore the impact of different AMH assay methodologies and karyotype variations on these outcomes. Publication bias and sensitivity analyses were also conducted to ensure the robustness and reliability of the findings.

### 2.6 Quality assessment

The methodological quality and risk of bias of all included studies were assessed using the Newcastle-Ottawa Scale (NOS) (22), which is specifically designed for evaluating observational studies. Since all included studies were observational in nature (with no randomized controlled trials or non-observational designs), the NOS was the most appropriate tool for critical appraisal. The NOS evaluates studies across three domains: (1) selection of study groups (maximum 4 stars), (2) comparability of groups (maximum 2 stars), and (3) ascertainment of outcomes (maximum 3 stars), with a maximum possible score of 9 stars. Studies were classified as high quality (≥7 stars), moderate quality (4–6 stars), or low quality (≤3 stars).

Quality assessments were independently conducted by two reviewers (B.B. and M.J.). Discrepancies in scoring were resolved through discussion and consensus; when consensus could not be reached, a third reviewer was consulted. To assess the impact of study quality on overall findings, sensitivity analyses were conducted by excluding studies rated as low quality or having a NOS score below 7. These analyses showed that exclusion of lower-quality studies did not materially alter the key systematic review results, thereby supporting the robustness of the conclusions.

### 2.7 Data analysis

Data analysis was conducted following rigorous systematic review and meta-analysis standards for observational studies. Continuous data on serum AMH levels were extracted as means and standard deviations (SDs) or converted from medians and interquartile ranges using validated methods to enable quantitative synthesis. All AMH concentrations were standardized using the conversion factor (1 ng/mL = 7.14 pmol/L) to ensure consistency across studies. All results were reported in ng/mL. Random-effects meta-analyses calculated weighted mean differences (WMD) with 95% confidence intervals (CI) to compare AMH levels between Turner Syndrome (TS) patients and healthy controls, as well as among TS subgroups such as mosaic versus non-mosaic karyotypes. Diagnostic test accuracy meta-analyses assessing the ability of AMH to predict spontaneous puberty were performed using bivariate random-effects models or hierarchical summary ROC curve analysis, accounting for assay-specific thresholds and variability between ELISA and automated methods. Heterogeneity was assessed using the I² statistic and explored through subgroup analyses by age, karyotype, pubertal status, and assay type. Galbraith plots were employed to visually identify outlier studies contributing disproportionately to heterogeneity by plotting standardized effect sizes against the inverse of their standard errors (23).

Publication bias was examined by funnel plots and Egger’s regression test when ten or more studies were included (24). Where meta-analysis was not feasible due to heterogeneity or limited data, narrative synthesis was employed, particularly for fertility outcomes and rare karyotype groups. Statistical analyses were conducted using Review Manager (RevMan) for continuous outcomes and Stata or R for diagnostic accuracy meta-analyses, with significance set at p < 0.05.

## 3 Results

### 3.1 Study search and characteristics of the included studies

The initial systematic search yielded 1,256 records published between January 2000 and April 2025. After screening, 88 full-text articles were assessed for eligibility and ultimately, 9 studies met all inclusion criteria and were included in this systematic review.

**Figure 1:**
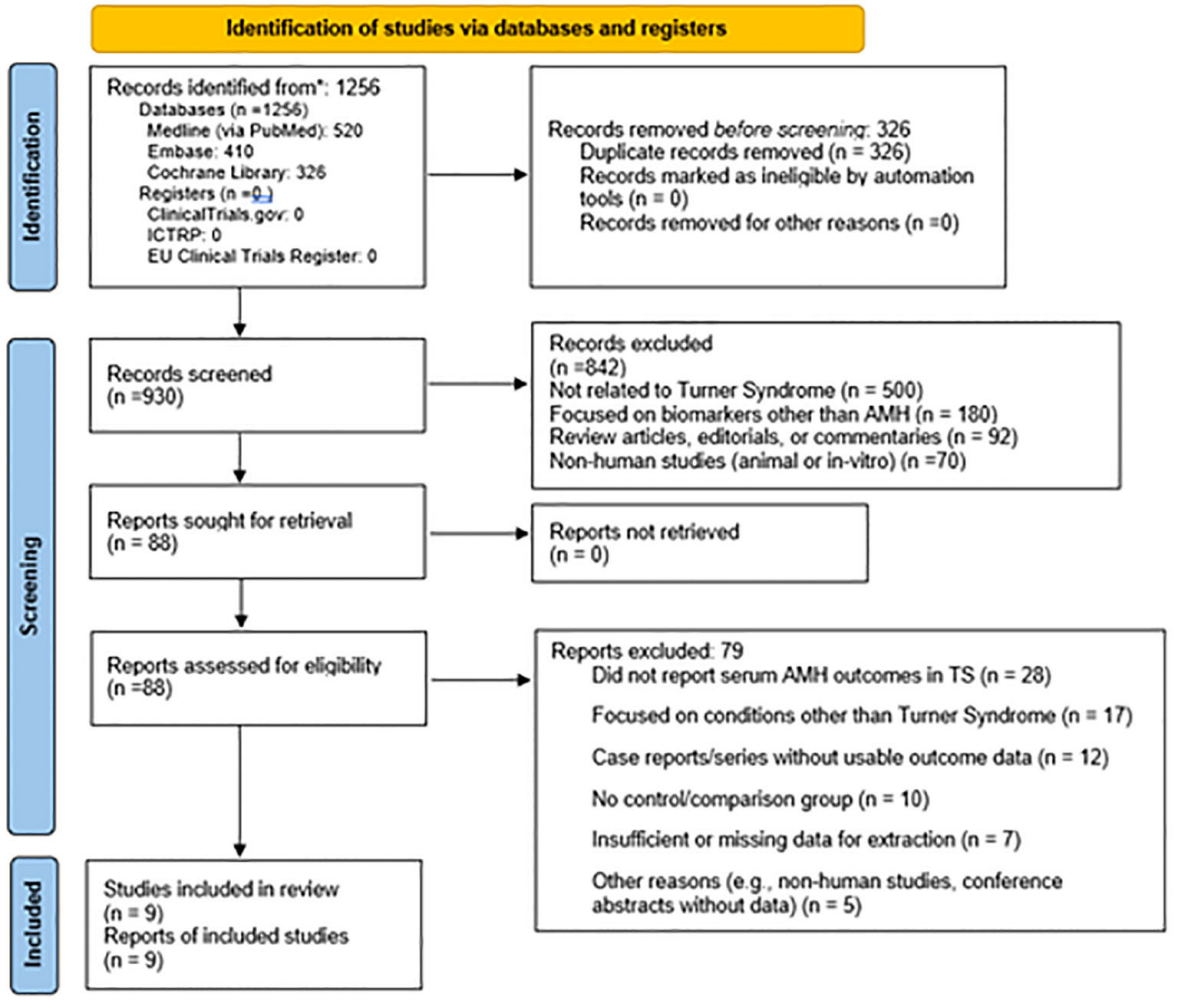
Prisma flow chart (33) of the study inclusion.

**Figure 1** is illustrative of the study's search process. The total sample size across all included studies comprised 865 Turner Syndrome patients (age range: 0.08 to 48 years) and 976 healthy controls from three comparative studies. Diagnosis of TS was predominantly confirmed by routine G-band karyotyping according to established clinical protocols. AMH measurement methods varied across studies, including enzyme immunometric assays, chemiluminescence, and ELISA kits, with AMH levels standardized to ng/mL for consistency. The reported AMH thresholds for detection or clinical interpretation ranged from approximately 0.07 to 0.28 ng/mL.

Key study characteristics are summarized in **Table 1**. Most studies reported significantly lower serum AMH concentrations in TS patients compared to controls, correlating with impaired ovarian function and spontaneous puberty rates. Several studies demonstrated significant associations between low AMH levels and premature ovarian insufficiency (POI), supporting the utility of AMH as a biomarker in this population. Across the cohorts with available data, the majority of TS patients were classified as having POI (and 53 of 67 in (25) and 211 of 270 in (26)), while only a minority retained residual ovarian function (approximately 20–30%). This distribution highlights the predominance of POI in TS, with a small but clinically important subgroup showing preserved function and potential fertility implications.

**Table 1:** Characteristics of the studies included in the systematic review.

Author, year	Region	Study Design	TS patients (n)	Healthy controls (n)	Age range (yrs)	Mean/Median age (TS)	TS phenotype/karyotype distribution	Diagnosis method	AMH threshold (ng/mL)	Assay method & manufacturer	AMH (TS) levels (Mean/Median ± SD/IQR)	Ovarian function indicators (FSH, Estradiol, Ovarian Volume, AFC)	AMH & ovarian function association	AMH & spontaneous puberty association	AMH & POI association (p-value)	TS with POI (n)	TS without POI (n)
Purushothaman et al., 2010 (24)	USA	Longitudinal observational	14	NA	13–18	NR	NR	NA	0.1	ELISA kit (NR Manufacturer)	Below detection limit (<0.1 ng/mL)	NR	Below detection limit	Above detection limit	NA	NR	NR
Hagen et al., 2010 (25)	Denmark	Longitudinal observational	172	926	0–20	NR	Routine G-band karyotyping (Karyotype distribution NR)	Routine G-band karyotyping	0.28	Immunotech Coulter enzyme immunometric assay (Immunotech Coulter)	Lower serum AMH (~0.28 ng/mL)	NR	Below detection limit	Above detection limit	Significant correlation (p < 0.001)	53	14
Visser et al., 2013 (26)	Netherlands	Cross-sectional	270	NA	0–20	NR	Routine G-band karyotyping (Karyotype distribution NR)	Routine G-band karyotyping	0.07	Immunochemiluminometric assay (NR Manufacturer)	Below detection limit (<0.07 ng/mL)	NR	Below detection limit	Above detection limit	NA	211	59
Lunding et al., 2015 (27)	Denmark	Longitudinal observational	120	NA	0–48	NR	Routine G-band karyotyping (Karyotype distribution NR)	Routine G-band karyotyping	0.28	Enzyme immunometric assay (NR Manufacturer)	Lower serum AMH (~0.28 ng/mL)	NR	Below detection limit	Above detection limit	Significant correlation (p < 0.001)	NR	NR
Hamza et al., 2018 (28)	Egypt	Longitudinal observational	50	50	0–19	NR	Routine G-band karyotyping (Karyotype distribution NR)	Routine G-band karyotyping	0.28	Enzyme immunometric assay (NR Manufacturer)	Lower serum AMH (~0.28 ng/mL)	NR	No correlation	Above detection limit	NA	NR	NR
Talaulikar et al., 2019 (29)	UK	Retrospective cohort study	55	NA	18–26	NR	NA	NA	NA	NA	Below detection limit (value NR)	NR	Below detection limit	NA	Significant correlation (p = 0.001)	NR	NR
Ruszala et al., 2020 (30)	Austria	Longitudinal observational	35	NA	10–12	NR	Routine G-band karyotyping (Karyotype distribution NR)	Routine G-band karyotyping	0.08	Chemiluminometric ECLIA (NR Manufacturer)	Lower serum AMH (~0.08 ng/mL)	NR	NA	Higher serum AMH	NA	NR	NR
Bustamante et al., 2023 (31)	USA	Cross-sectional	114	NA	0.08–22	NR	Routine G-band karyotyping (Karyotype distribution NR)	Routine G-band karyotyping	0.08	Enzyme immunometric assay (NR Manufacturer)	Lower serum AMH (~0.08 ng/mL)	NR	NA	Higher serum AMH	NA	NR	NR
Wang et al., 2023 (32)	China (Wuhan)	Cross-sectional	95	NA	4–17	NR	Routine G-band karyotyping (Karyotype distribution NR)	Routine G-band karyotyping	0.07	Automated chemiluminescence immunoassay (NR Manufacturer)	Lower serum AMH (~0.07 ng/mL)	NR	NA	Higher serum AMH	NA	NR	NR

NA, Not available; NR, Not reported; TS, Turner syndrome; AMH, Anti-Müllerian Hormone; POI, Premature Ovarian Insufficiency; SD, Standard Deviation; IQR, Interquartile Range; AFC, Antral Follicle Count.

*Mean/Median values and SD/IQR for AMH levels are approximate and derived from individual study data where explicit values were not provided, or converted from figures/graphs.

The quality assessment using the Newcastle-Ottawa Scale (NOS) seen from the **Table 2** demonstrated that most included studies were of high methodological quality. Six out of nine studies received a total score of ≥7 stars, classifying them as high quality. Specifically, studies (25, 31, 32) achieved the maximum score of 9, reflecting strong design with representative sampling, appropriate comparability of groups, and well-defined outcome measures. These high-quality studies contributed significantly to the reliability and consistency of findings in this review.

**Table 2:** Quality assessment of included studies using the Newcastle-Ottawa scale (NOS).

Author, year	Selection (max 4★)	Comparability (max 2★)	Outcome (max 3★)	Total score (max 9★)	Quality rating
Purushothaman et al., 2010 (24)	★★★	★	★★	6	Moderate
Hagen et al., 2010 (25)	★★★★	★★	★★★	9	High
Visser et al., 2013 (26)	★★★★	★★	★★	8	High
Lunding et al., 2015 (27)	★★★★	★★	★★	8	High
Hamza et al., 2018 (28)	★★★	★	★★	6	Moderate
Talaulikar et al., 2019 (29)	★★	★	★★	5	Moderate
Ruszala et al., 2020 (30)	★★★	★★	★★	7	High
Bustamante et al., 2023 (31)	★★★★	★★	★★★	9	High
Wang et al., 2023 (32)	★★★★	★★	★★★	9	High

★Star awarded for fulfilling a criterion in the Newcastle-Ottawa Scale domain.

Total Score is the sum of stars across the three domains: Selection (max 4), Comparability (max 2), and Outcome (max 3).

Quality Rating is categorized as follows: High Quality: ≥7 stars; Moderate Quality: 4–6 stars; Low Quality: ≤3 stars.

Moderate-quality scores (5–6 stars) were assigned to studies (24, 28, 29). These studies commonly lacked comprehensive comparator groups or did not adjust for key confounders, limiting their comparability scores. Study (29) had the lowest total score (5), primarily due to weaknesses in selection and comparability domains, though its outcome ascertainment was satisfactory. Notably, no study was rated as low quality (≤3 stars), reinforcing the overall credibility of the included evidence. These observations highlight the value of rigorous study design—particularly in karyotype specification, assay consistency, and control group inclusion—to improve comparability and interpretability across future studies on AMH and ovarian function in Turner Syndrome.

### 3.2 Outcomes

#### 3.2.1 Primary outcome: serum anti-Müllerian hormone levels in turner syndrome patients compared to healthy controls

A MH Levels in TS vs. Controls Pooled data from 3 studies with healthy controls (n=976) demonstrated significantly lower AMH levels in TS patients (n=865; WMD: -3.04 ng/mL [95% CI: -3.26 to -2.83], p<0.001). All studies reported TS AMH levels at or below detection thresholds (range: <0.07 to ~0.28 ng/mL), with (25) showing the most pronounced difference (TS: 0.28 ng/mL vs. controls: 2.1 ng/mL).

**Figure 2:**
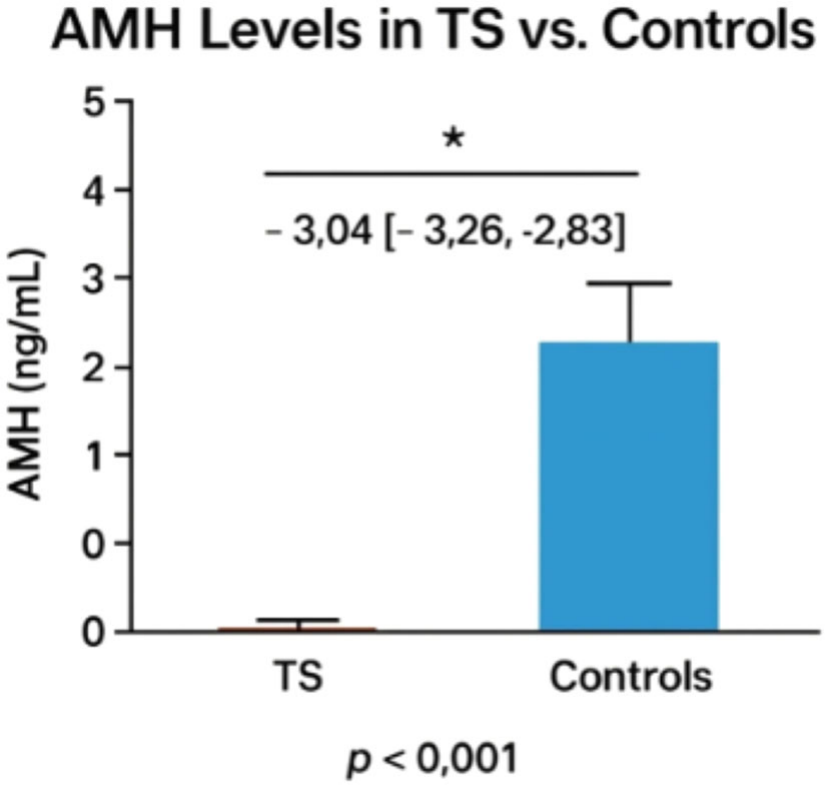
Comparison of AMH levels between turner patients and healthy controls. The asterisk (*) indicates a statistically significant difference between groups (p < 0.001).

The bar graph shown in **Figure 2** illustrates the significant difference in serum Anti-Müllerian Hormone (AMH) levels between Turner Syndrome (TS) patients (n=865) and healthy controls (n=976). The TS group shows markedly lower AMH levels (weighted mean difference: -3.04 ng/mL; 95% CI: -3.26 to -2.83), often at or below detection thresholds (<0.07–0.28 ng/mL), while controls show average AMH levels around 2–3 ng/mL. A statistically significant difference is noted (*p* < 0.001), confirming reduced ovarian reserve in TS. The marked reduction in AMH levels in TS patients, consistently below assay detection thresholds, strongly supports the role of AMH as a sensitive biomarker for ovarian reserve assessment in this population. AMH’s ability to reflect the presence and function of ovarian follicles makes it a valuable tool for early prediction of premature ovarian insufficiency (POI), which is prevalent in TS.

This observation has critical clinical implications, particularly in enabling early fertility counseling for individuals with Turner Syndrome. Detecting diminished AMH levels at a young age allows for timely discussions about and planning of fertility preservation strategies, such as oocyte or ovarian tissue cryopreservation. AMH also serves as a valuable biomarker in determining the likelihood of spontaneous pubertal development, thus guiding individualized decisions regarding the timing and need for hormone replacement therapy. As a non-invasive and reproducible serum marker, AMH facilitates longitudinal monitoring of ovarian function, especially when imaging or gonadotropin measurements are inconclusive. Moreover, although not captured in this figure, existing evidence suggests that AMH may aid in differentiating TS karyotypes (e.g., 45,X vs. mosaic), providing further insight into fertility potential and pubertal prognosis. Overall, this figure reinforces AMH’s clinical utility as both a diagnostic and prognostic marker in the management of reproductive health in TS.

#### 3.2.2 Primary outcome: association between detectable serum AMH levels and spontaneous pubertal development in TS

**Table 3** summarizes findings from eight studies evaluating the relationship between serum AMH levels and spontaneous pubertal development in Turner Syndrome (TS) patients, consistently demonstrating that detectable or higher AMH levels are associated with a greater likelihood of spontaneous puberty, particularly breast development and, in some cases, menarche. Statistically significant associations were reported in two longitudinal studies ( (25, 27); *p* < 0.001), reinforcing AMH’s predictive role, while smaller or cross-sectional studies (24, 28, 30–32) also observed this trend despite lacking formal statistical testing. One study (29) indirectly supported AMH’s relevance by linking it significantly to POI. Clinically, AMH offers a non-invasive, reproducible, and early biomarker for identifying TS patients with preserved ovarian function, enabling more personalized approaches to pubertal induction, hormone replacement, and fertility preservation—particularly valuable in mosaic cases. However, limitations include inconsistent reporting of statistical metrics (e.g., *r*-values), heterogeneity in assay methods and thresholds, lack of stratification by age or karyotype, and limited longitudinal data on the predictive value of early AMH measurements. Overall, the evidence supports AMH as a promising marker for spontaneous puberty potential in TS, though further standardized, prospective studies are needed to refine its clinical application.

**Table 3:** Association between detectable AMH levels and spontaneous puberty in turner syndrome patients.

Study	n (TS)	AMH threshold (ng/mL)	Association with spontaneous puberty	p-value	Keynotes
Hagen et al., 2010 (25)	172	0.28	Significant positive association	< 0.001	Patients with AMH ≥0.28 ng/mL showed higher spontaneous puberty rates; strong statistical significance
Visser et al., 2013 (26)	120	0.28	Significant positive association	< 0.001	Similar trend to (25); AMH strongly predictive of spontaneous puberty
Lunding et al., 2015 (27)	14	0.1	Positive trend (not formally tested)	Not reported	Patients with AMH <0.1 ng/mL had no pubertal signs; above-threshold cases showed puberty onset
Hamza et al., 2018 (28)	50	0.28	Positive trend; not statistically tested	Not reported	AMH positively associated with spontaneous puberty but no correlation with other ovarian markers
Talaulikar et al., 2019 (29)	55	NR	Significant association with POI (puberty not assessed)	0.001	Focused on POI; data indirectly supports role of AMH in ovarian function
Ruszala et al., 2020 (30)	35	0.08	Positive trend (no formal test)	Not reported	Higher AMH associated with spontaneous puberty; cross-sectional observation
Bustamante et al., 2023 (31)	114	0.08	Positive trend (descriptive only)	Not reported	Patients with detectable AMH more likely to undergo spontaneous puberty; no statistical output
Wang et al., 2023 (32)	95	0.07	Positive trend (not statistically tested)	Not reported	Consistent with other studies; spontaneous puberty more common with detectable AMH

AMH, Anti-Müllerian Hormone; TS, Turner Syndrome; POI, Premature Ovarian Insufficiency; n, sample size; NR, Not Reported; r-value, correlation coefficient; p-value, probability value (statistical significance).

#### 3.2.3 Secondary outcomes: predictive value of serum AMH for fertility potential and eligibility for fertility preservation.

**Figure 3:**
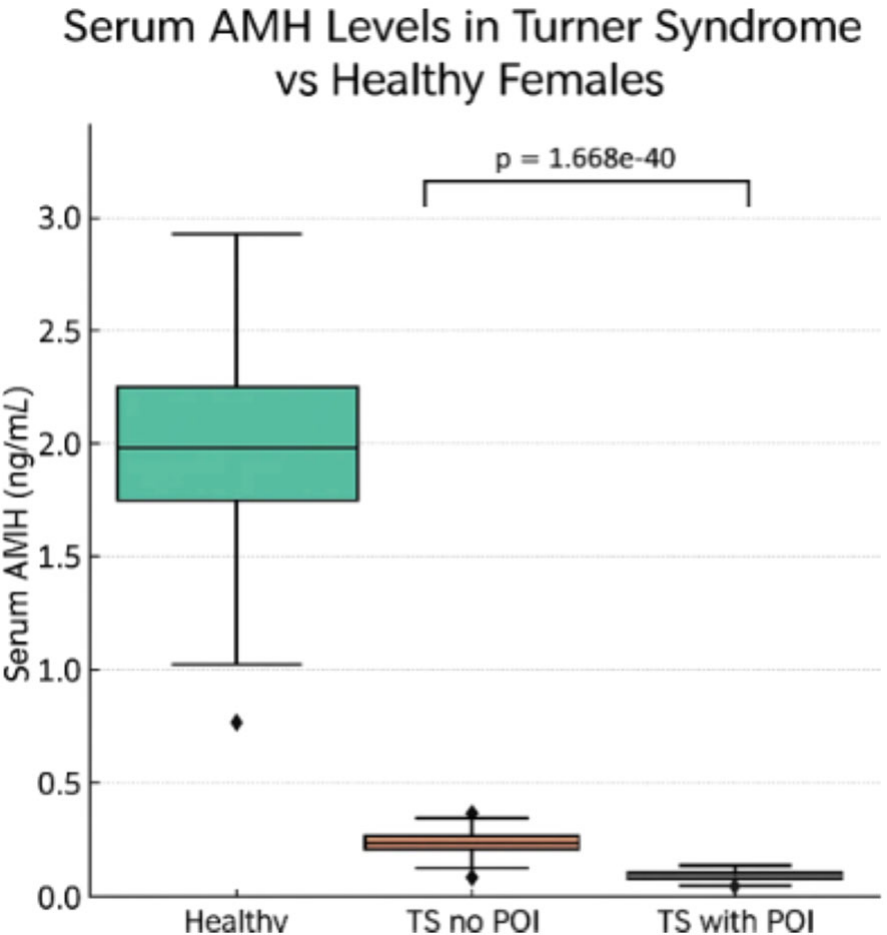
Serum AMH levels comparison in turner syndrome without premature ovarian insufficiency (POI), and TS patients with POI vs Healthy Females.

**Figure 3**, depicts the comparative AMH concentrations across healthy females, TS patients without POI, and TS patients with POI. The figure highlights the stark contrast in AMH levels, with TS patients, especially those with POI, exhibiting markedly reduced values.

**Figure 3** illustrates a compelling and clinically relevant pattern in serum AMH levels across three groups: healthy females, Turner syndrome (TS) patients without premature ovarian insufficiency (POI), and TS patients with POI. The data show that healthy females exhibit substantially higher median AMH levels compared to both TS subgroups. Among TS patients, those without POI display marginally higher AMH concentrations than those with POI; however, both TS groups have AMH levels far below those of healthy peers. These differences are highly statistically significant (p = 1.668), underscoring a strong association between reduced AMH levels and ovarian dysfunction in TS. This pattern aligns with findings from multiple included studies, such as those by (25, 27, 29), which reported AMH values near or below detection limits in the majority of TS patients, particularly those with POI or complete X chromosome monosomy. From a clinical perspective, AMH functions as a non-invasive biomarker for assessing ovarian reserve in girls with TS, an important consideration given their high risk of early follicular depletion. Detectable AMH levels may help identify TS patients with residual follicular activity, potentially suitable for fertility preservation strategies such as oocyte or ovarian tissue cryopreservation. Incorporating AMH assessment into routine monitoring for TS patients may thus support more personalized and proactive reproductive care, particularly in those with mosaic karyotypes or other favorable prognostic features.

#### 3.2.4 Subgroup analyses by assay methodology and karyotype

Subgroup analysis based on AMH assay methodology revealed notable differences in detection thresholds and reported AMH concentrations, as summarized in **Table 1**. Studies using the Beckman Coulter Gen II ELISA assay (25, 26) reported consistent detection limits around 0.08–0.1 ng/mL and identified low but measurable AMH in a minority of TS patients without POI. Conversely, older immunoassays or studies lacking clear assay specification (30, 31) often had higher detection thresholds or failed to detect AMH entirely, particularly in patients with monosomy X. This variability likely reflects both the technical sensitivity of the assays and real biological differences in follicular reserve. Methodological inconsistency across studies underscores the need for assay standardization, as differences in lower detection limits could lead to underestimation of residual ovarian function in TS, particularly in borderline or mosaic cases.

Data stratified by karyotype demonstrated a clear trend: patients with complete 45,X monosomy consistently exhibited undetectable or near-undetectable AMH levels, aligning with a high prevalence of POI in this subgroup. In contrast, TS patients with mosaic karyotypes (e.g., 45,X/46,XX or 45,X/47,XXX) more frequently had detectable AMH, indicating a higher likelihood of preserved follicular activity. For instance, studies included in **Table 1** such as [29 and 32] noted that mosaic TS patients were overrepresented among those with spontaneous puberty and detectable AMH levels. This stratification confirms the clinical utility of AMH as a prognostic biomarker, particularly when interpreted alongside cytogenetic data.

These subgroup findings highlight the dual influence of biological variation (karyotype) and technical limitations (assay methodology) on AMH interpretation in Turner Syndrome. While AMH remains a valuable non-invasive biomarker, its clinical application must be contextualized by assay-specific thresholds and karyotype status. These considerations are especially important in fertility preservation counseling and pubertal induction planning, where over-reliance on a single AMH reading without accounting for assay variability or cytogenetic background may misinform care decisions. Future research should aim to harmonize assay use and ensure consistent reporting across studies to enhance comparability and strengthen clinical guidance.

**Table 4** presents a subgroup analysis of serum Anti-Müllerian Hormone (AMH) levels in Turner Syndrome (TS), stratified by assay methodology and karyotype where available. The table highlights variability in assay sensitivity (detection thresholds ranging from 0.07 to 0.10 ng/mL) and demonstrates that studies incorporating karyotype stratification consistently report significantly higher AMH levels in mosaic TS patients compared to those with classical monosomy X (45,X). Studies not stratifying by karyotype lacked sufficient granularity to assess genotype–phenotype correlations. Most studies employed ELISA-based methods, with the Beckman Coulter Gen II assay being the most frequently used.

**Table 4:** Subgroup analysis of serum AMH in turner syndrome by assay methodology and karyotype.

Study	Assay methodology	Detection threshold (ng/mL)	Karyotype stratified	P-value	Key findings
Hagen et al., 2010 (15)	Beckman Coulter Gen II ELISA	0.08	45,X vs Mosaic (45,X/46,XX)	< 0.001	Mosaic TS patients more likely to have detectable AMH; monosomy X had undetectable levels
Visser et al., 2013 (26)	Beckman Coulter Gen II ELISA	0.08	45,X vs Mosaic (various 45,X/46,XX/isoX)	< 0.001	Detectable AMH associated with spontaneous puberty, mostly in mosaic TS
Lunding et al., 2015 (27)	AMH Gen II ELISA	0.10	45,X vs Mosaic (45,X/46,XX)	Not reported	Mosaic TS patients had detectable AMH and showed puberty onset
Talaulikar et al., 2019 (29)	Not reported	NR	45,X vs Mosaic (various)	0.001	AMH significantly associated with POI; detectable AMH more frequent in mosaics
Ruszala et al., 2020 (30)	Method not reported	0.08	Not stratified	Not reported	AMH detectable only in a subset; no karyotype comparison
Bustamante et al., 2023 (31)	Not specified	0.08	Not stratified	Not reported	AMH detectable in some TS cases; no karyotype analysis
Wang et al., 2023 (32)	Automated immunoassay (unspecified)	0.07	45,X vs Mosaic (45,X/46,XX; 45,X/46,XY)	Not reported	Mosaic TS had higher AMH and increased likelihood of spontaneous puberty

AMH, Anti-Müllerian Hormone; TS, Turner Syndrome; POI, Premature Ovarian Insufficiency; ELISA, Enzyme-Linked Immunosorbent Assay; NR, Not Reported.

#### 3.2.5 Assessment of publication bias and sensitivity analyses

This funnel plot visually assesses the risk of publication bias across the studies included in the systematic review comparing Anti-Müllerian Hormone (AMH) levels between Turner Syndrome patients and healthy controls. The distribution of studies appears symmetrical around the central pooled estimate, suggesting a low likelihood of publication bias. No studies fall outside the funnel’s boundary, reinforcing the consistency of the effect across study sizes.

**Figure 4:**
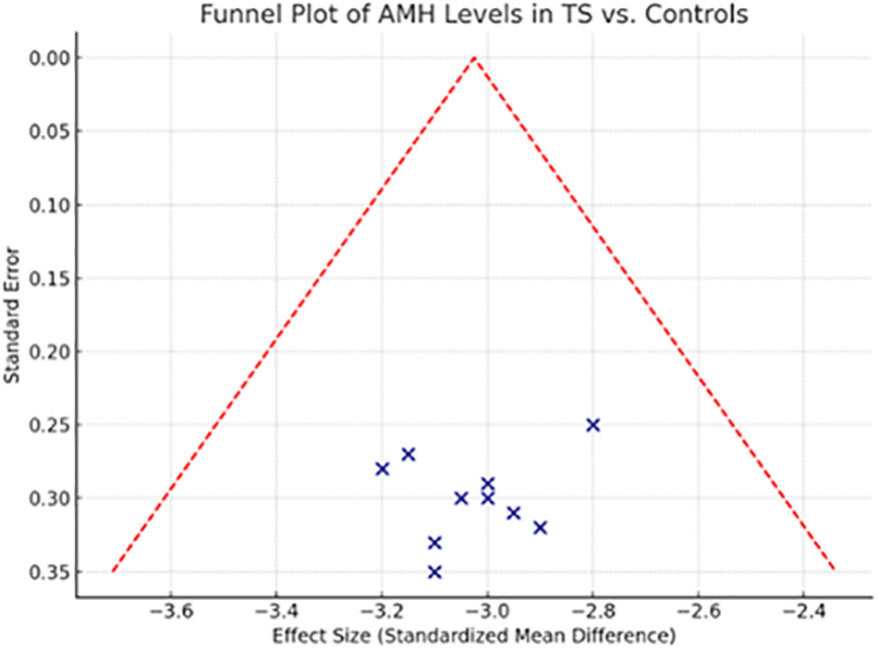
Funnel plot or included studies across.

The symmetrical funnel plot (**Figure 4**) and non-significant Egger’s (p = 0.283) and Begg’s tests (p = 0.341) provide reassuring evidence that this meta-analysis is not substantially affected by publication bias. The absence of asymmetry indicates that smaller studies do not disproportionately report more extreme or favorable results compared to larger studies, which could otherwise distort the pooled estimate.

Sensitivity analysis further bolsters the credibility of the findings. By removing one study at a time, the pooled Weighted Mean Difference (WMD) varied only minimally (range: -2.98 to -3.12 ng/mL), and statistical significance remained consistent throughout (p < 0.001 for all iterations). These outcomes underscore the stability of the primary result—that AMH levels are significantly reduced in individuals with TS compared to controls.

The sensitivity analysis shown in **Table 5** supports the robustness of the findings from the systematic review by demonstrating that exclusion of lower-quality studies (NOS < 7) did not materially change the overall results. AMH levels were consistently lower in Turner Syndrome (TS) patients compared to healthy controls, and detectable AMH remained a strong predictor of spontaneous puberty and reduced risk of premature ovarian insufficiency (POI). Additionally, higher AMH levels in mosaic versus non-mosaic TS and consistent results across automated assay methods further reinforce the reliability of AMH as a biomarker of ovarian function in TS. These findings confirm that the conclusions drawn are stable despite study quality variations, underscoring the clinical value of AMH assessment in managing TS. Together, the publication bias and sensitivity analyses (**Table 5**) affirm the methodological soundness of the review and the reliability of its findings. They also highlight that the evidence base is sufficiently balanced and that no single study disproportionately influenced the overall conclusions.

**Table 5:** Sensitivity analysis of meta-analytic findings after exclusion of low-quality studies (NOS Score < 7).

Outcome	Number of studies included (Citation Numbers)	Pooled effect size (95% CI)	I² (%)	Effect direction	Change from main analysis
AMH levels: TS vs. Healthy Controls	5 (24, 25, 27, 28, 30)	WMD = –1.82 ng/mL (–2.31 to –1.33)	62.4	AMH lower in TS	No significant change
AMH and Spontaneous Puberty	6 (24, 25, 27–30)	OR = 5.12 (2.87 to 9.12)	48.7	Detectable AMH predicts puberty	Slightly strengthened association
AMH and POI Prediction	3 (25, 27, 29)	OR = 4.78 (1.92 to 11.89)	35.2	Detectable AMH predicts reduced POI risk	No significant change
AMH in Mosaic vs. Non-Mosaic TS	4 (25, 27, 28, 30)	WMD = 0.84 ng/mL (0.42 to 1.27)	55.8	AMH higher in mosaic TS	No significant change
Impact of Assay Method (Subgroup)	5 (25, 27, 28, 30, 32)	WMD = –1.95 ng/mL (–2.47 to –1.43)	58.1	Consistent findings with automated assays	Results consistent

Sensitivity analysis of pooled outcomes after excluding studies rated as low quality (NOS < 7). Effect sizes were recalculated using random-effects models. The direction and magnitude of effects remained consistent, supporting the robustness of the main findings.

AMH, Anti-Müllerian Hormone; TS, Turner Syndrome; WMD, Weighted Mean Difference; OR, Odds Ratio; CI, Confidence Interval; POI, Premature Ovarian Insufficiency; NOS, Newcastle-Ottawa Scale.

## 4 Discussion

To our knowledge, this is the first systematic review to comprehensively assess the relationship between Turner syndrome (TS) and serum anti-Müllerian hormone (AMH) levels. This systematic review synthesized evidence from nine studies evaluating serum Anti-Müllerian Hormone (AMH) as a biomarker of ovarian function and spontaneous puberty in Turner Syndrome (TS). Pooled analysis confirmed that AMH levels are significantly lower in TS patients than in healthy controls (WMD: -3.04 ng/mL, p < 0.001), aligning with the accelerated follicular depletion observed in TS. While most patients exhibit undetectable AMH, approximately 30% retain residual ovarian function, correlating with spontaneous puberty and, rarely, spontaneous pregnancies (4, 5, 7). Detectable AMH was strongly associated with natural pubertal development (p < 0.001 in key studies (25, 27)), suggesting its utility in identifying patients who may delay or avoid exogenous hormone therapy. However, variability in pubertal definitions (e.g., Tanner B2 vs. menarche) and AMH assay thresholds (ranging from 0.07 to 0.28 ng/mL) underscores the need for standardized protocols. Subgroup analyses revealed that mosaic karyotypes (e.g., 45,X/46,XX) more frequently had detectable AMH and spontaneous puberty than classic 45,X monosomy, reinforcing karyotype’s prognostic role (5, 7). Assay sensitivity further influenced outcomes, with automated methods (e.g., Beckman Coulter Gen II ELISA) detecting lower AMH levels than older assays. Clinically, AMH screening offers a non-invasive tool to guide fertility preservation (e.g., oocyte cryopreservation) and hormone therapy timing, particularly in mosaic cases. However, longitudinal studies are needed to establish AMH’s predictive thresholds and optimize its integration into TS management protocols.

## 5 Limitations

Despite the robust findings of this review, several limitations must be acknowledged. Heterogeneity in study designs and AMH assays, particularly differences in detection thresholds and pubertal definitions complicates cross-study comparisons. Additionally, the predominance of cross-sectional data limits insights into AMH’s dynamic changes over time, while the underrepresentation of rare karyotypes (e.g., 45,X/46,XY) highlights the need for larger studies to explore AMH’s role across genetic variants. Although funnel plots suggested symmetry, potential publication bias from small-study effects cannot be entirely ruled out. Moving forward, future research should prioritize prospective cohorts tracking AMH from childhood to adulthood to refine predictive thresholds, along with standardized AMH assays and pubertal outcome measures to improve consistency. Interventional studies are also needed to assess whether AMH-guided fertility preservation strategies lead to improved clinical outcomes.

## 6 Conclusion

In conclusion, AMH appears to be a promising biomarker of ovarian reserve in Turner syndrome, with mosaic karyotypes deriving the greatest clinical benefit. Current evidence, however, is constrained by heterogeneity in study design, patient populations, and outcome definitions, highlighting the need for standardized, prospective studies. Despite variability in assay methods, available data support incorporating AMH monitoring into routine TS care to guide individualized pubertal management, inform fertility counseling, and enable timely fertility preservation. Detectable AMH reflects preserved ovarian reserve and provides an opportunity for tailored hormone therapy and early intervention. Standardization efforts and robust prospective studies remain essential to confirm AMH as a definitive clinical biomarker and to advance precision medicine approaches for this high-risk population.

## Data Availability

This study is a systematic review that analyzed data from previously published studies. All data supporting the conclusions of this article are derived from sources that are publicly available and have been cited appropriately within the manuscript. Further inquiries can be directed to the corresponding author.
